# Chronic Diffuse Nephritis without Exudation

**Published:** 1897-03

**Authors:** Joe S. Wooten

**Affiliations:** Austin, Texas


For the Texas Medical Journal.

### CHRONIC DIFFUSE NEPHRITIS WITHOUT EXUDATION—A REPORT OF CASES.

BY JOE S. WOOTEN, B.SC., M.D., AUSTIN, TEXAS.

EVER since John Bright, in 1827, first pointed out the intimate association existing between certain cases of dropsy and albuminuria on the one hand, and morbid structural and functional changes in the kidneys on the other, the study of the pathology and symptomology of renal diseases, and its relations to, and influence exerted on, other morbid changes in the body, has never ceased to interest and excite the mind of the profession. In honor of the distinguished investigator, the term "Bright's disease" has ever since been employed to embrace all forms of inflammatory changes in the kidneys attended by albuminuria and dropsy. In the light of more modern pathological observations, however, we have been forced to recognize and acknowledge, clinically, at any rate, a form of kidney affection in which these two symptoms play, at best, a very minor part, and afford us an uncertain aid in diagnosis. As did the announcement of the discovery by Bright and his cotemporaries—that constant albuminuria and casts were pathognomonic of kidney disease—profoundly disturb clinical medicine, so, too, did the positive declaration by Delafield, Jacobi—among children, and others, that the obverse of the above rule was not true, but that the dead-house and the laboratory had corroborated suspicions long held, and conclusively demonstrated to them the frequent existence of advanced inflammatory alterations in the kidney in the absence of the triad of symptoms which go to make up the more common history of "Bright's disease." It would be irrelevant to the subject matter of my paper to recite here the details of such researches, or to give at length the etiology and morbid anatomy of nephritis in general. Suffice it to say, that much of the valuable information and light which have been thrown upon the all important subject of chronic productive and exudative inflammation in general, have come through investigations along this line, and a wide step made towards placing the practice of medicine beyond the realm of empiricism onto a more scientific, pathological basis. It would be better, from a scientific point of view, if the term "Bright's disease" could be discarded from our nomenclature; but the term has probably come to stay, and generically used, it is still retained as a convenient form for grouping together a variety of kidney affections. Probably it would not be out of place, then, for me to first briefly enumerate here the divisions of such an accepted classification (Delafield and Prudden, Histological and Pathological Anatomy), and I do it for a better understanding, and in order that you might see just where, in the table this atypical form of nephritis—of which I would speak to-day—belongs.

I.—Acute Bright's disease:—

(1) Acute congestion of the kidney.

(2) Acute degeneration of the kidney.

(3) Acute exudative nephritis.

(4) Acute productive nephritis.

II.—Chronic Bright's disease:—

(1) Chronic congestion of the kidney.

(2) Chronic degeneration of the kidney.

(3) Chronic productive (diffuse) nephritis with exudation.

(4) Chronic productive (diffuse) nephritis *without exudation.*

Vigorous objections have been and will yet be raised against such an array of classification, but the day in medicine has passed, in which one can scientifically speak and write in glittering generalities. Doubtless criticism will be passed upon the act of subdividing the chronic productive type into an exudative and a *non-exudative form*, on the grounds that it is a useless classification, a distinction without a difference. While it is true, the lesion in the main in both is essentially the same—a chronic inflammation with the production of new connective tissue in the stroma of the kidney—so, too, in other inflammatory diseases, the pleuræ for example—where the inflammatory changes *per se* are the same, nevertheless, the resulting products vary, and we have an accepted and rational classification, (1) of pleurisy—with the production of fibrin, (2) pleurisy—with the production of serum and fibrin, (3) pleurisy—with the production of serum, fibrin and pus (empyema), with a distinct clinical picture common to each. And likewise, in chronic interstitial nephritis does the clinical picture vary according to the presence or absence of albumin in the urine.

In a classical case of the exudative type of interstitial nephritis, a mistake in diagnosis is hardly conceivable; the anæmia, the œdema, the cerebral and cardiac disturbances and polyuria, co-existing probably with arterios-clerosis,—would lead us at once to make the diagnosis of "Bright's disease," and we would proceed to confirm the opinion by a hasty chemical examination of the urine for the detection of albumen, and a subsequent assumption of the presence of casts. Not many years ago, in the failure to find albumen in the urine of a suspicious case, there would have been very few physicians who would not have unhesitatingly pronounced the "kidneys sound." Urine of a low specific gravity, containing albumen and casts, has been for years familiar to even the tyro as being diagnostic of nephritis; but the physician who would have announced then the coexistence of grave renal lesions in a patient passing urine of a normal specific gravity, with absence of albumen, and—less frequently—of casts, would have been—and was later—laughed at. Nevertheless, such a state of things is possible, and late researches have shown it of far greater frequency than is commonly suspected.

The five cases, reports of which will follow, have been examples encountered during the short period of a year. The first three present more forcibly the fact which I desire most to lay stress upon, and which led me to contribute this paper—i. e., the atypical form of symptoms which often accompany chronic nephritis, for it is the strange and unusual types of a disease which most interest us in medicine, and not the easily differentiated and classical cases.

CASE 1. ——, male, single, age 32, a laborer. Previous history and habits good. Had scarlet fever when sixteen, and la grippe about four years ago. Previous to this spell, in 1891, was enjoying fair health, but since then has never had the same constitution or felt his old self again. Has complained more or less of cardiac pain, oppression and palpitation; has been subject to "asthmatic spells" for the past eight or nine years, with intervals of two to six months between attacks. Complains now of basal headaches, sudden attacks of blindness, dyspnœa on exertion, rheumatism and constipation; suffers at times from pain over lumbar region, and with polyuria. Sleeps soundly, except when disturbed by tumultuous action of heart. There is often gastric distress after eating. Has never had dropsy, puffiness of eyelids nor swollen feet. Consulted physician always for heart trouble, and has never had kidneys pronounced unsound. Physical condition on first day of examination was as follows: Anæmia; general artero sclerosis; pulmonary emphysema; cardiac hypertrophy, more especially of left heart, apex beat displaced downward and to the left, and distinctly visible; the left ventricular border about one-eight inch beyond mammary line; an aortic regurgitant murmur, with compensatory changes apparently complete. Urine:—*first examination*—clear, highly colored, and strongly acid, sp. gr. 1.025; *no albumen;* no sugar; hyaline and granular casts. Second examination, slightly cloudy, normally colored and normally acid, sp. gr. 1.018, with a trace *of albumen*, hyaline, granular and epithelial casts, white and red blood cells.

CASE 2. ——, male, single, age 65, a German, and a blacksmith by trade. Previous history good. Has used alcoholics all his life, but never to excess. About one year and a half ago, patient began to notice a partial failure in eyesight; consulted an oculist, and while the resulting ophthalmoscopic examination proved suspicious, yet after the negative results of two chemical examinations, made by a druggist, the idea of albumino-retinitis was discarded. The vision in both eyes has steadily grown worse, until now there is only light perception and a condition of advanced nerve atrophy. Has had polyuria for years, being often obliged to get up several times during a night. Has never had dropsy, and but very little puffiness about eyes and ankles, and only this of late. Has been subject for past six years to occasional attacks of violent headache, vomiting and fever, all of which he took to be "bilious" in character. Is troubled at times with dyspnoea, cardiac pain and distress. The extreme waxy appearance of skin first led me to suspect nephritis. Physical examination: Extreme anæmia; general arterio-sclerosis; left cardiac hypertrophy, apex beat visible and displaced downward and to the left, the left ventricular border extending well beyond mammary line; heart sounds dull, muffled and prolonged, no murmur distinguishable. Urine, on several examinations, very clear, of light color, acid; sp. gr. varying from 1.008–1.017; *no albumen;* hyaline casts plentiful, but few granular and epithelial. In one of the examinations, *neither albumen nor casts to be found.*

CASE 3. ——, female, age 26; married. Family history good. Previous to about two years ago had enjoyed good health, but since then has been a sufferer from female trouble, with its train of symptoms . . . . and which caused her to consult a physician. None of the subjective or physical symptoms presented were other than what one would expect to find accompanying similar local trouble, but the anuria, sometimes polyuria, the persistent and severe headaches, the palpitation and dyspnoea, with now and then suspicious puffing about the eyes, and slight swelling of ankles, led to close and repeated examinations of the patient's urine. Urine:—First examination—sp. gr. 1.025, normally acid and of light color; a trace of albumen; hyaline, granular and epithelial casts and cell detritus. In all, five examinations were made at different times. In three of them, *no albumen*, but casts were found; in another, *neither albumen nor casts;* in the last, a trace of albumen, casts and blood cells. The sp. gr. varied from 1.011–1.030.

CASE 4. ——, male, age 57, and married. A family history of hæmophilia. Has been a steady drinker for years. Has never had rheumatism. Previous to five years ago was in fairly good health, except for the symptoms hereafter given, then after an attack of la grippe, first began to notice serious trouble; remained anæmic, debilitated and weak; there was palpitation and dyspnœa on exertion. Has had for the past fifteen years, a hemorrhagic tendency; bleeds easily from scratches, and without cause, from the nose and gums. During the past ten years has suffered from alternating attacks of gastritis, diarrhœa and constipation, with slight jaundice. Has never had ascites. Since the la grippe, all of these symptoms have proven more frequent and severe, and the patient has steadily lost flesh and strength. In addition, he suffers from severe headaches, sudden attacks of blindness, dyspnœa, precordial oppression, and tumultuous heart's action lasting from a few minutes to several hours. Has never lost consciousness. Vomits without nausea. During the past few years has noticed that the kidneys have acted more frequently, and has been obliged to get up two or three times at night. The polyuria is most noticeable after one of the above mentioned attacks. Previous to one year ago, had never observed any puffiness of the eyes or swollen ankles; lately his attention has been been called to this. On my examination, his physical condition was as follows: anæmia; a waxy color; slight thickening of the arteries; a cirrhotic liver; splenic enlargement; hypertrophy and dilatation of heart, the apex beat being two inches below and almost on nipple line, and the left ventricular border about one-fourth inch beyond its normal position. The heart sounds were dull, muffled and prolonged; there was present a diffused, aortic regurgitant murmur. Urine: normally acid and of normal color; sp. gr. 1.014; *no albumen*, many hyaline and some few granular casts. On several subsequent examinations, no albumen was found, always hyaline casts, the epithelial variety being found only after diligent search. Sp. gr. varied, 1.012-1.020.

CASE 5. ——, male, age 62, married; a farmer. Family history good. Has had chronic rheumatism for past ten years, but never confined to his bed. Has been a drinker all his life. For a number of years has had sharp, shooting pains through the heart; palpitation; dyspnœa accompanied at times with severe cyanosis, severe occipital headaches and vertigo; has never had dropsy or swollen feet; has suffered at times with severe pain in the region of both kidneys. During the past year and a half, all these symptoms have grown worse; the attacks of dyspnœa come while lying down, and the cardiac and cerebral symptoms irrespective to exertion or excitement; For a long time had difficulty in passing his urine; the quantity was small and highly colored; now there is polyuria and the urine is clear and like water. About six months ago noticed some swelling of ankles, which came and went, but has steadily grown worse. Physical examination was as follows: Pronounced anæmia and a waxy color; marked artero-sclerosis; puffed lids and œdema of extremities. There was marked cardiac hypertrophy; its action was irregular and tumultuous; there was a loud aortic regurgitant murmur, and what I took to be a mitral stenotic (diastolic) murmur as well. Urine of sp. gr. 1.008, acid and almost colorless; no albumen, but granular cast in abundance, with some few of the epithelial variety. Strange to say, the patient was able to ride several miles horseback to town, and appeared not to be especially affected by the exertion.

In case I, then, we have, in all probability, an example of the effect of an acute infectious disease on the kidneys, producing primarily either an acute parenchymatous degeneration, or an acute productive nephritis, which terminated or continued on into a chronic productive form. In case II, we have an illustration of a common occurrence, a primary chronic congestion produced by the long continued use of alcohol, terminating into a chronic inflammation with the production of new tissue. In case four, we have the above two causes combined, the inflammatory changes taking place first in the liver and finally in the kidneys. Contrary to what is generally stated to be the case when the two diseases—cirrhosis of the liver and the non-exudative nephritis—are combined, there was no dropsy. In all of the cases except one, there was hypertrophy of the left ventricle. The acid, the heat and the aceto-ferrocyanide tests were employed in examiniug for albumen. To avoid destruction of tube casts from the decomposition of urea, all deposits were examined within two hours after collecting urine specimen, and were stained either by the boro-carmine or eosin method.

It is unnecessary here to enter into a consideration of the various theories which have been advanced in explanation of the physiological secretion and elimination of urine. Suffice it to say, that the Conheim-Herdenhain theory is the one most generally accepted, that the secretion of urine is not alone a mechanical process depending upon a relative degree of blood pressure, but in addition—upon a special selective action of the renal epithelia, and increases or diminishes, cæteris paribus, with the dilation of the renal vessels and the resulting current rate. But a word as to the cause and conditions which produce albuminuria, before first considering the special features of this type of nephritis characterized by its absence. "The weight of evidence of most observers tends to prove that albuminuria occurs whenever in the kidney the rapidity of the blood current falls below that which is necessary for the nourishment and life of the epithelium covering the glomerli. Whenever this state occurs, whenever these epithelia receive less oxygen and less of the other elements necessary for the maintenance of their physiological integrity than they require, then they become permeable to the albumen of the serum of the blood, which filtering through, becomes mixed with the watery elements that form the urine." Following up this line of reasoning, transient albuminuria has been explained—in part—on the same hypothesis by supposing a state of tetanic contraction of the arterioles of the kidney sufficient to produce temporary anæmia, and thereby to impair the nutrition and disturb the function of the epithelium; or else, as a result of hyperæmia and stasis from other causes, an analogous condition of disturbed nutrition is brought about, permitting a transudation of serum, an emmigration of white blood cells and a diapedesis of colored blood cells.

Now, first, why is it that in this form of nephritis we, as a rule, find no albumen? Aside from supposing it to be primarily a form of chronic non-exudative inflammation, there occurred to me other causes which operate against its presence. In this type of nephritis, especially, do we find compensatory hypertrophy of the left ventricle, owing to the increased and prolonged action of intra renal resistance to the circulation. Some of the same causes which predispose to this ventricular hypertrophy, the slow development and extreme chronicity of the disease, also play their role here. The encroachment, involvement and obliteration of the glomeruli and tubules by thickenings, new cell infiltrations and tissue growths, take place slowly; but few glmeruli are affected at a time, the diseased tufts and tubules are shut off or stop secreting, and the remaining healthy portions of the kidney do the work. It is only during the exacerbations of the disease, and the advancement of the productive inflammatory lesion on to new grounds,—as is shown by the coexistence of other inflammatory products,—that albumen make its appearance at all in the urine. Second, why is it that dropsy is not present, or becomes a late symptom in the disease? As a result of the compensatory processes—in form of a hypertrophied ventricle and an increased blood pressure, together with the presence of many remaining healthy glomeruli and uriniferous tubules, the kidneys still do their work, and the patients continue to pass, as a rule, a normal quantity of urine; or else there is an excess, and, owing to the disproportion between the solid and watery elements, of a low sp. gr. and of very pale color. And to this normal or excessive secretion and elimination of the solid and watery elements of the urine, do we, in part, explain the absence of the œdema, except as a late symptom, and then the result of a cardiac failure. Hence, it is that chronic uræmia, too, is not a frequent symptom. Acute uræmia, however, is; we must therefore conclude that there are other factors at work in acute uræmia not to be found in the chronic form, and that the difference between them is not alone founded on the absence, duration or the occurrence of prodromal symptoms, or alone to the accumulation of urotoxæmic products in the blood; for Delafield has shown: (1) that these cerebral symptoms often develop in patients, who are passing large quantities of urine, of fair specific gravity; (2) and are again entirely absent in other cases with a diminished secretion of urine; (3) that by acute uræmia we mean those cerebral and circulatory symptoms attended upon a contraction of the arteries (be the cause what it may), accompanied by sudden dyspnoæ, twitchings or convulsions, vomiting, delirium, stupor or sudden coma, high temperature, and a tense pulse; (4) that by the administration of arterial dilators, these symptoms are at first often averted or relieved. (Student Lectures, College of Phys. and Surg.)

There is no question in my mind but what many of the reported cases of so-called idiopathic swelling or œdema of the extremities, and a certain per centage of cases with cerebro vasomotor disturbances and nervous symptoms appearing in men of middle and advanced life, are but examples of this form of non-exudative nephritis. The centrifuge has made many revelations, and will yet work many changes in our diagnosis.

[Since first contributing this paper, there have come under my observation three new cases of this type of nephritis, but whose clinical picture varies little from the essential symptoms above given, and are, therefore, not detailed here.]

CONCLUSIONS.

1. That the microscopical test is underestimated, and too little practiced.

2. That the chemic test as a means to a diagnosis of chronic nephritis is misleading, and not to be relied upon.

3. That albumen without the presence of casts is rare, but casts in the absence of albumen is a very frequent occurrence.

4. That there may be a cyclical occurrence of casts, and between times even a microscopical examination proves nil.

5. That the typical low sp. gr. of nephritic urine is often absent, and hence the fallacious logic of reasoning from the sp. gr. as to the condition of kidneys, and the line of urinary examination necessary.

6. The necessity in these cases of suspecting a nephritis from objective and subjective symptoms alone.

7. The necessity of fresh specimens of urine, of stained deposits, and of repeated examinations.

